# Supplementary material for: Optimizing cell therapy by sorting cells with high extracellular vesicle secretion
Source: Nat Commun. 2024 Jun 7;15:4870. doi: 10.1038/s41467-024-49123-1 (PMC11161503; doi:10.1038/s41467-024-49123-1)
Supplement: Supplementary file 1 — Supplementary Information [file 41467_2024_49123_MOESM1_ESM.pdf]

Supplementary Information for:

Optimizing cell therapy by sorting cells with high extracellular vesicle secretion

Authors: Doyeon Koo[1*], Xiao Cheng[2*], Shreya Udani[1], Sevana Baghdasarian[1], Dashuai Zhu[3], Junlang Li[4], Brian Hall[5], Natalie Tsubamoto[1], Shiqi Hu[3], Jina Ko[6,7], Ke Cheng[3†], Dino Di Carlo[1,8,9,10†]

Affiliations:
[1]Department of Bioengineering, University of California, Los Angeles; Los Angeles, CA 90095, USA.
[2]Joint Department of Biomedical Engineering, University of North Carolina at Chapel Hill and North Carolina State University; Chapel Hill, NC 27599, and Raleigh, NC 27607, USA.
[3]Department of Biomedical Engineering, Columbia University; New York, NY 10032, USA.
[4] Xsome Biotech; Raleigh, NC 27606, USA
[5]Cytek Biosciences; Fremont, CA 94538, USA.
[6]Department of Pathology and Laboratory Medicine, University of Pennsylvania; Philadelphia, PA 19104, USA.
[7]Department of Bioengineering, University of Pennsylvania; Philadelphia, PA 19104, USA.
[8]Jonsson Comprehensive Cancer Center, University of California, Los Angeles; Los Angeles, CA 90095, USA.
[9]Department of Mechanical and Aerospace Engineering, University of California, Los Angeles; Los Angeles, CA 90095, USA.
[10]California NanoSystems Institute; Los Angeles, CA 90095, USA.
*These authors contributed equally to this work.
†Corresponding authors. Email: dicarlo@ucla.edu, ke.cheng@columbia.edu

Supplementary Information List:

Supplementary Figure 1: Nanovial fabrication and functionalization.

Supplementary Figure 2: Optimization of single-cell EV secretion assay using nanovials.

Supplementary Figure 3: Regrowth of cells following sorting based on EV secretion levels.

Supplementary Figure 4: Transcriptomic analysis of MSC markers, EV biogenesis and stem cell proliferation expression.

Supplementary Figure 5: Differences in tissue regeneration signature expression between high and low secretors.

Supplementary Figure 6: Gene ontology annotations for top 20 upregulated genes in high secretors.

Supplementary Figure 7: Analysis and isolation of mouse MSCs based on EV secretion level.

Supplementary Figure 8: High secretors exhibit higher potential to reduce cell apoptosis following H2O2-induced mouse cardiomyocyte (H9c2 cells) injury.

Supplementary Figure 9: High EV-secreting MSCs show augmented ability to mitigate cardiac remodeling after MI.

Supplementary Figure 10: Survival rate of mice treated with high and low EV secreting MSCs.

Supplementary Figure 11. Survival rate of mice treated with high and low EV secreting MSCs.

Supplementary Table 1. Individual electrocardiography data of the experimental mice.

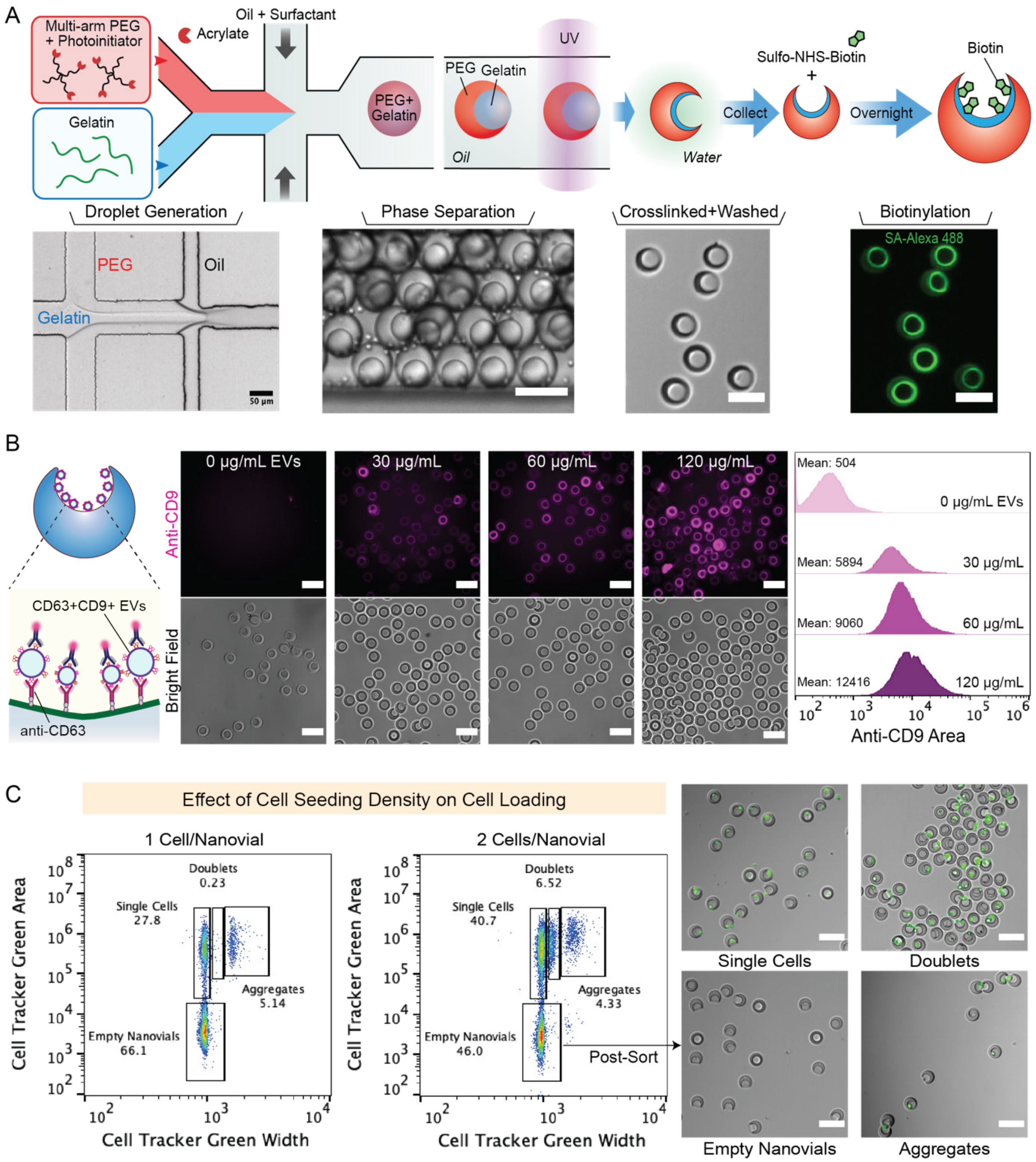

Supplementary Figure 1. Nanovial fabrication and functionalization. A) An aqueous phase consisting of 4-arm-polyethylene glycol (PEG) acrylate and photo-initiator is co-flowed with a gelatin solution in a microfluidic droplet generator. After droplet formation, PEG and gelatin undergo phase separation forming a PEG-rich surrounding phase and gelatin-rich internal phase. The PEG outer structure is cross-linked by exposure to UV light. The gelatin phase is washed away except for a layer that is cross-linked at the interface of the cavity. After collection, nanovials are incubated with sulfo-NHS-biotin to biotinylate the internal gelatin-coated cavity. Localized fluorescence of AlexaFluor488-labeled streptavidin is observed in biotinylated nanovial cavities. Scale bar represents 50 μm. B) Schematic of the EV capture assay shows the anti-CD63 capture

and fluorescent anti-CD9 detector antibodies. Flow cytometry fluorescence histograms and fluorescence microscopy images of nanovials following exposure to increasing amounts of EVs. Nanovials were functionalized with anti-CD63 antibodies and incubated with 0, 30, 60, or 120 µg/mL of isolated EVs from the conditioned medium. Scale bars represent 100 µm. C) Gating strategy for flow cytometry analysis and sorting of single cells on nanovials. Flow cytometry scatter plots, gates, and microscopy images of gated events are shown. The highest fraction of single-cell loaded nanovials was achieved when cells were seeded at 1 cell per nanovial. Nanovials with single cells were sorted based on the fluorescent area vs. width signal of cells (cell tracker green). Scale bars represent 100 µm. Panels A and B created with BioRender.com, released under a Creative Commons Attribution-NonCommercial-NoDerivs 4.0 International license.

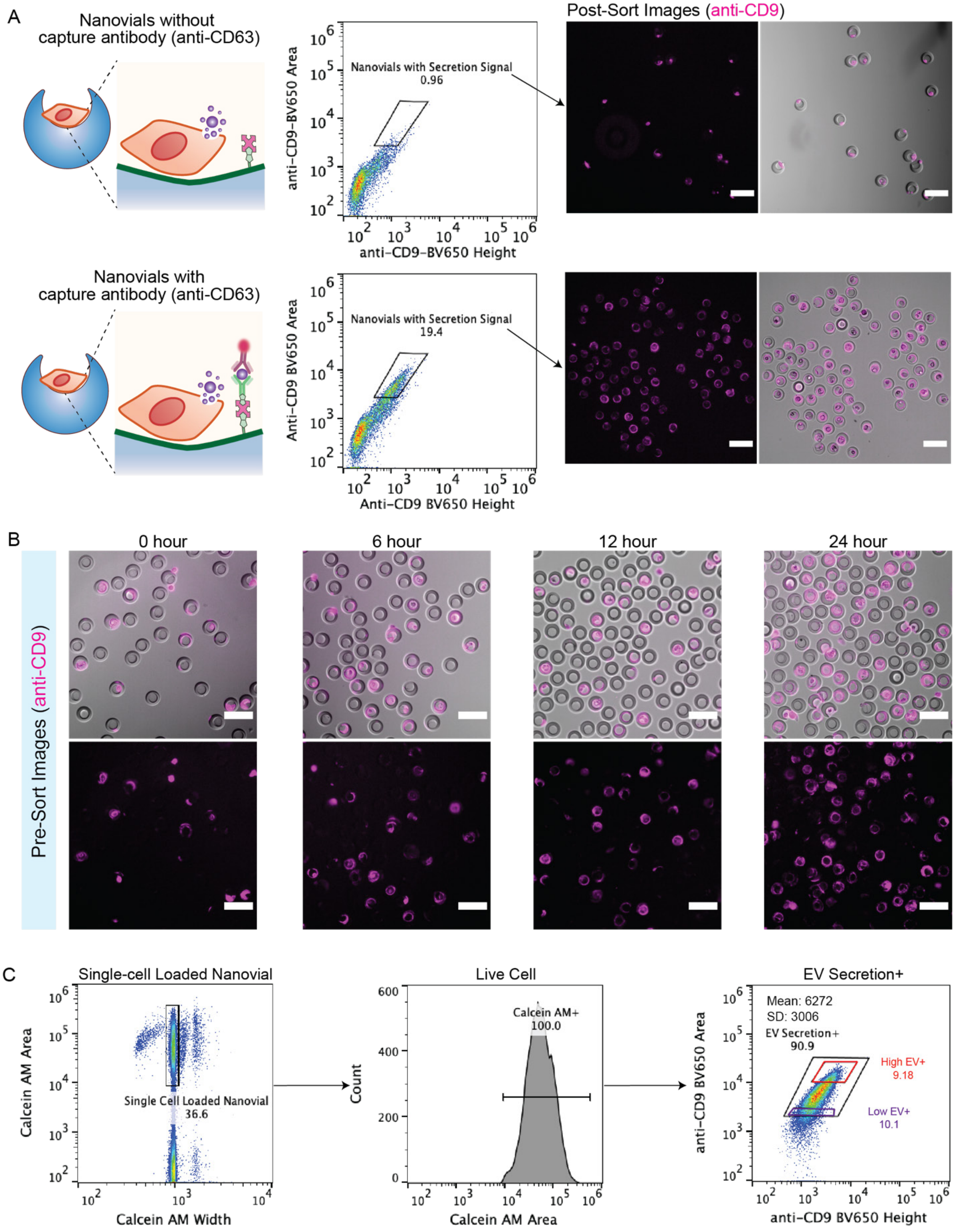

Supplementary Figure 2. Optimization of the single-cell EV secretion assay using nanovials. A) Flow cytometry analysis of EV secretion after 24 hours of secretion accumulation on cell-loaded

nanovials. Strong CD9 signal was observed from cells loaded onto nanovials with anti-CD63 EV capture antibody, while secretion was not detected from cells loaded onto nanovials without capture antibody. Fluorescence microscopy images showing post-sort population from the illustrated “secretion positive” gate are shown. Scale bars represent 100 μm. B) Fluorescence microscopy images of EV secretion signals on cell-loaded nanovials after 0 to 24 hours. The highest CD63+CD9+ EV secretion signal was observed when secretion was accumulated over 24 hours on nanovials. Scale bars represent 100 μm. C) Flow cytometry flows showing gating of single-cell loaded nanovials with positive calcein AM staining For viable cells, significant heterogeneity in secretion is observed. Single live cells on nanovials spanned secretion signals over one order of magnitude with coefficient variance of 0.49. Panel A created with BioRender.com, released under a Creative Commons Attribution-NonCommercial-NoDerivs 4.0 International license.

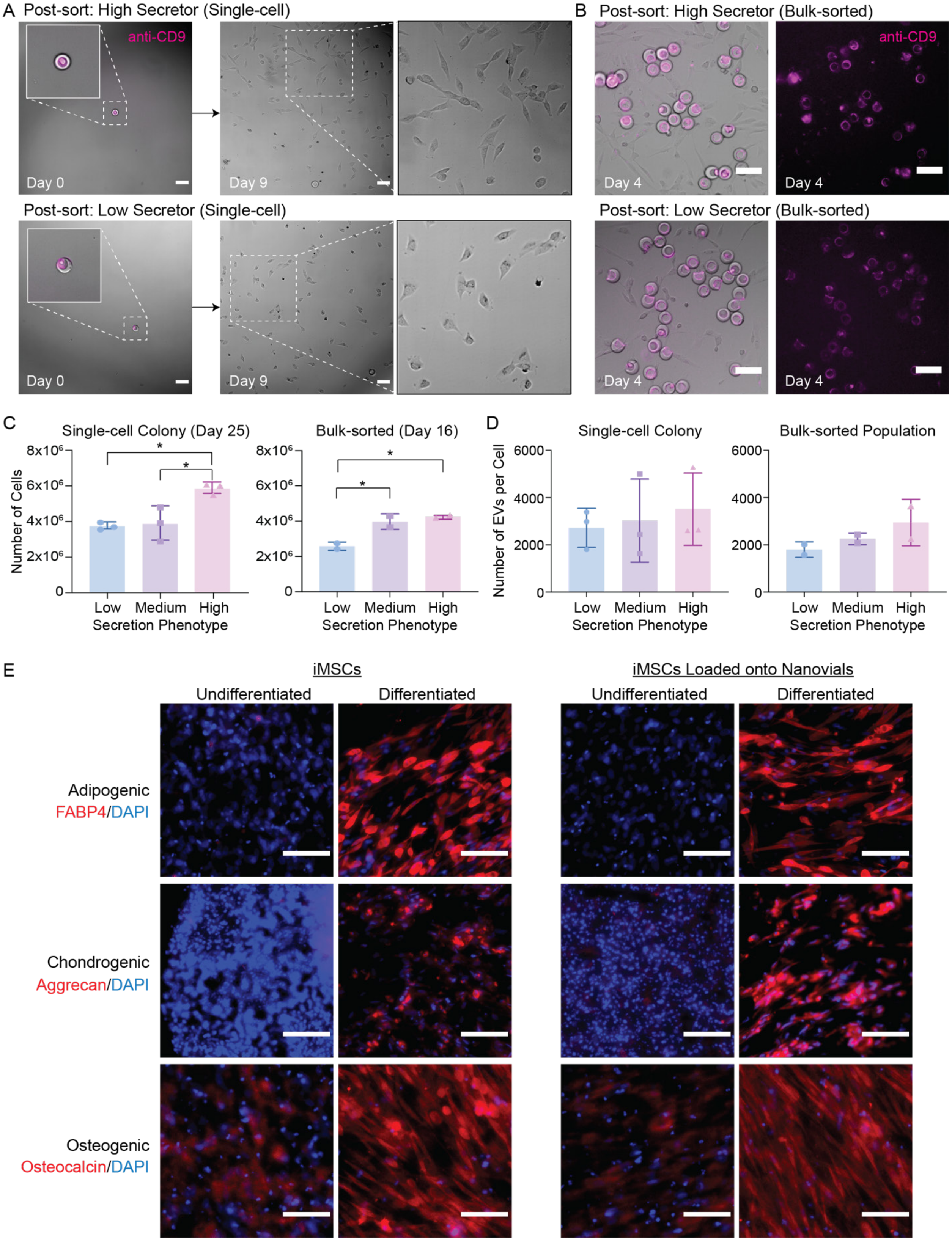

Supplementary Figure 3. Regrowth of cells following sorting based on EV secretion levels. A) Expansion of single-cell colonies from high and low EV secretors shown from Day 0 to Day 9.

Scale bars represent 100 μm. B) Expansion of bulk-sorted cells (3000 cells) sorted based on secretion level on Day 4. Secreted EVs are still retained inside the cavity of nanovials at Day 4. Scale bars represent 100 μm. C) Final number of cells in a colony expanded from single cells on Day 25 or bulk-sorted cells on Day 16. Increased proliferation was observed from the high secretors in both single-cell colonies and bulk-sorted populations. Significant difference claimed by one-way ANOVA with post-hoc Tukey Honestly Significant Difference (HSD) test (*$p<0.05$). D) EV production rate (EVs/cell) from single-cell colony and bulk-sorted populations quantified from conditioned media. n=3 for single-cell colony and n=2 for bulk-sorted colony. E) Multi-potency characterization of iMSCs and iMSCs loaded into nanovials. iMSCs loaded on nanovials were sorted, expanded and induced for differentiation. Original iMSCs that were never loaded onto nanovials were also sorted and induced differentiation as a negative control. Representative immunofluorescence images (20X magnification) of adipogenic, osteogenic, and chondrogenic differentiation assays. Cells were stained with NorthernLights 557-conjugated donkey anti-goat (adipogenic or chondrogenic) or donkey anti-mouse (osteogenic) secondary antibodies (red) and the nuclei were counterstained with DAPI (blue). Scale bars represent 100 μm.

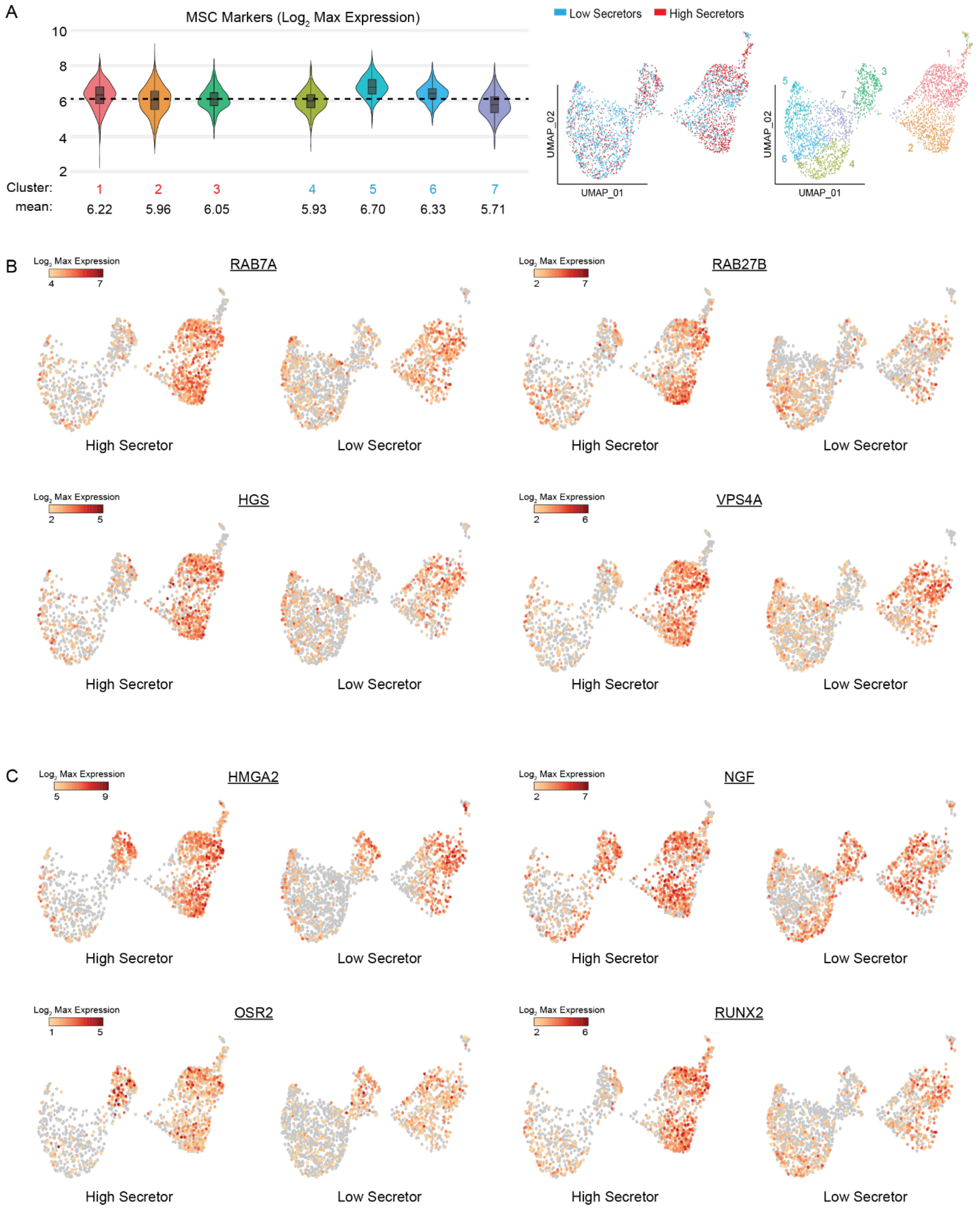

Supplementary Figure 4. Transcriptomic analysis of MSC marker and EV biogenesis expression. A) MSC marker expression was consistent within each cluster, indicating the nanovials do not affect intrinsic characteristic of stem cells. B) Genes associated with EV biogenesis (RAB7A, RAB27B, HGS, VPS4A) or C) with positive regulation of stem cell proliferation (HMGA2, NGF, OSR2, RUNX2) are specifically overexpressed among high secretors.

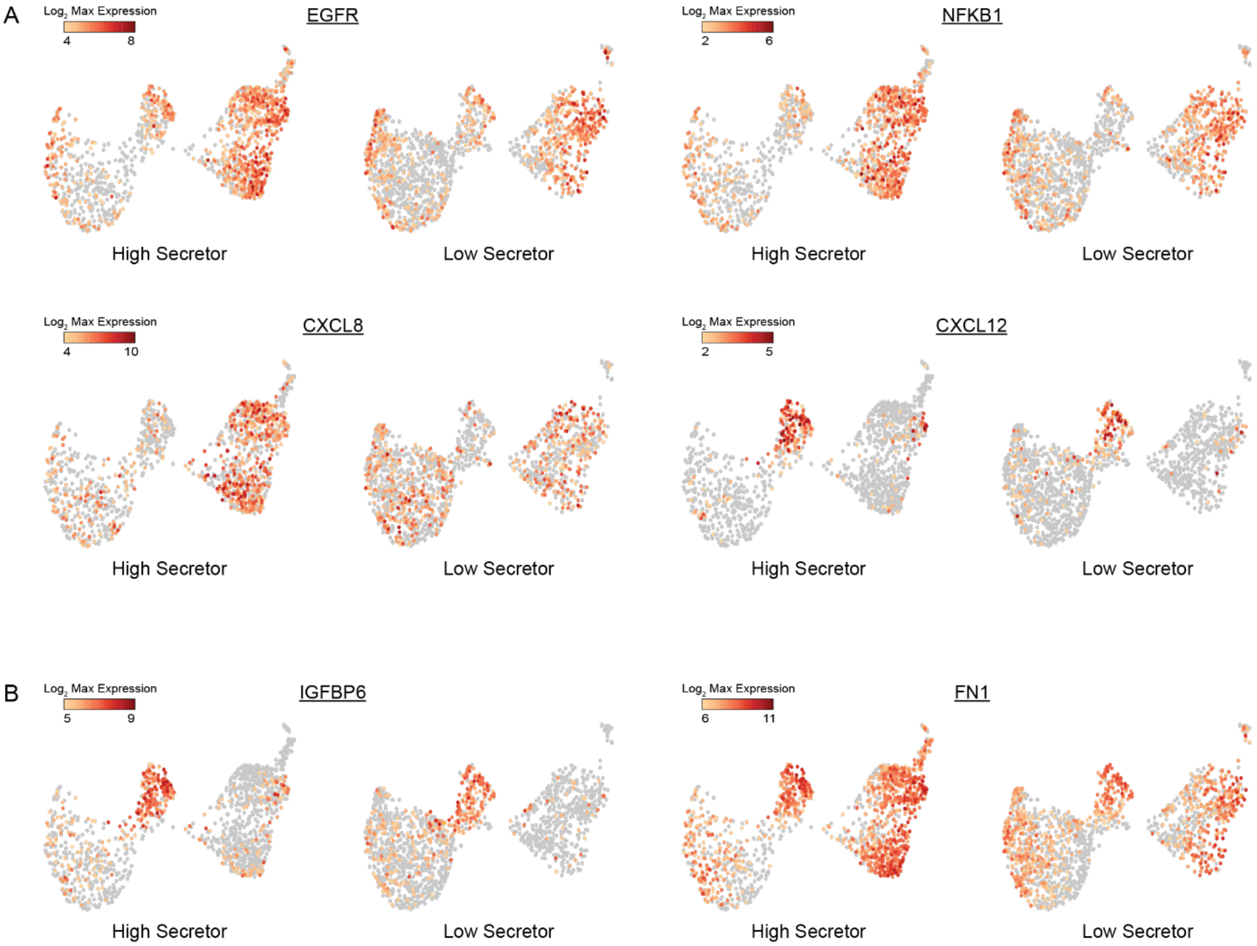

Supplementary Figure 5. Differences in expression level associated with tissue regeneration and vascular regenerative signal between high and low secretors. A) Pro-angiogenic factors such as EGFR, NFKB1, CXCL8, and CXCL12 are overexpressed among high secretors. B) Vascular regenerative signal associated genes such as IGFBP6 and FN1 are specifically expressed in cluster 3 and high secretors exhibited greater expression of these genes.

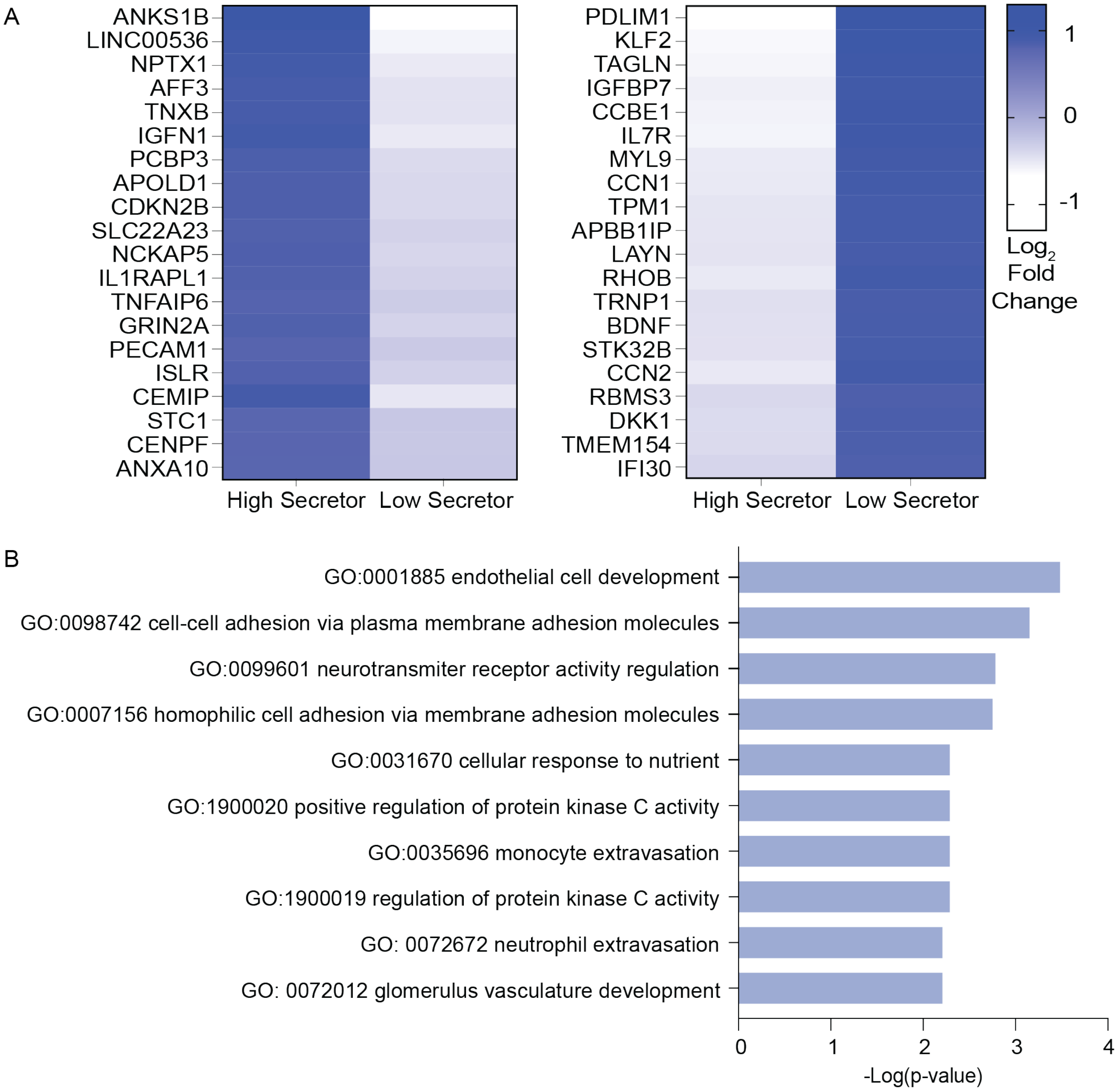

Supplementary Figure 6. Top 20 differentially expressed transcripts for high and low secretors and ontology terms associated with genes upregulated in high secretors. A) Distinct mRNA expression profiles in high vs. low EV secretors. The inclusion criteria of these transcripts was a 2-fold difference of log2 (fold-change) with a *p*-value <0.05. Blue signal, represents higher relative expression as compared to light blue signal. B) GO Biological Process annotations using the Enrichr toolkit for multiple testing with a *p*-value <0.01 (FDR<0.05) identified terms associated with the top 20 genes upregulated among high secretors.

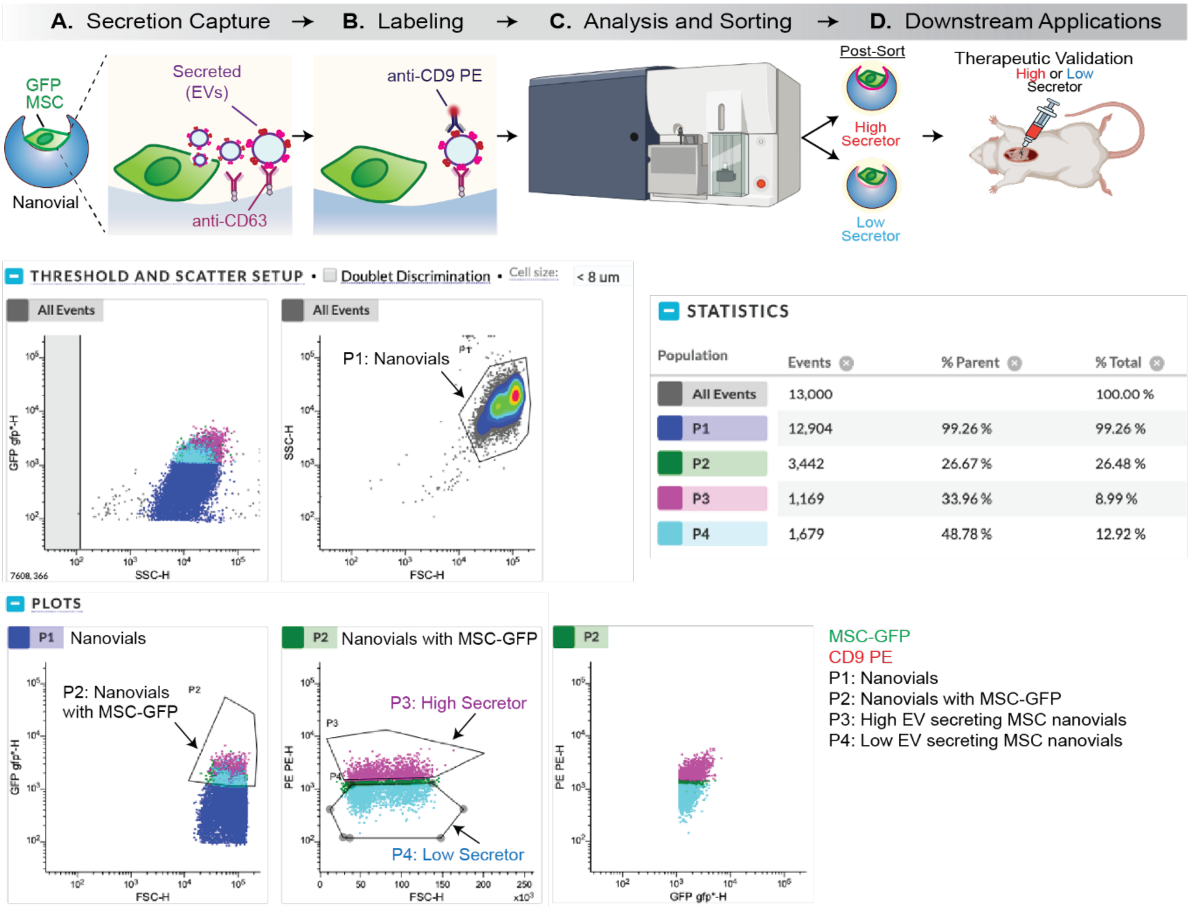

Supplementary Figure 7. Analysis and isolation of mouse MSCs based on EV secretion level. Schematic of the assay for mouse cells. Schematic was created with Biorender.com. Cells are loaded on anti-CD63 nanovials and sorted based on anti-CD9 PE signal along with cell marker (GFP).

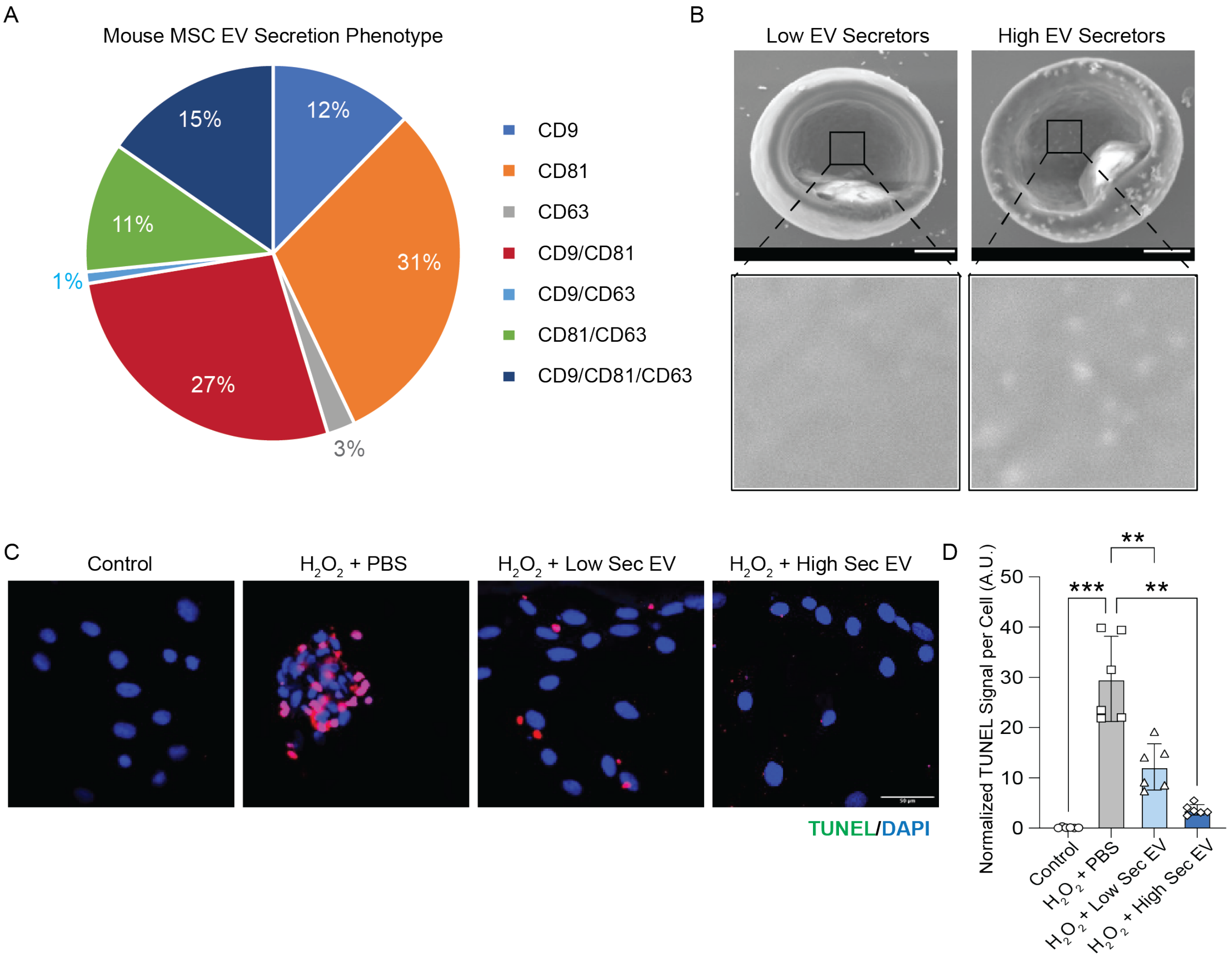

Supplementary Figure 8. Mouse MSC EV secretion characteristic and their therapeutic potential. A) Tetraspanin abundance for EVs secreted by mouse MSCs analyzed by ExoView. B) Scanning electron microscopic images showing single mouse MSC loaded nanovial and captured EVs on the nanovial surface after sorting cells based on high and low EV secretion signal. Scale bar represents 5 µm. C) EVs from high-sec MSCs exhibit a higher potential to reduce cell apoptosis following H_2O_2-induced mouse cardiomyocyte (HL-1 cells) injury represented by TUNEL staining of cells. Scale bar represents 100 µm. D) Quantification of apoptosis of cells (TUNEL+). (n=6, biological replicates). Comparisons between more than two groups were performed using the one-way analysis of variance (ANOVA), followed by Tukey's honestly significant difference (HSD) post hoc test. The comparisons between samples are indicated by lines, and the statistical significance is indicated by asterisks above the lines. $^{**}p < 0.01$, $^{***}p < 0.001$.

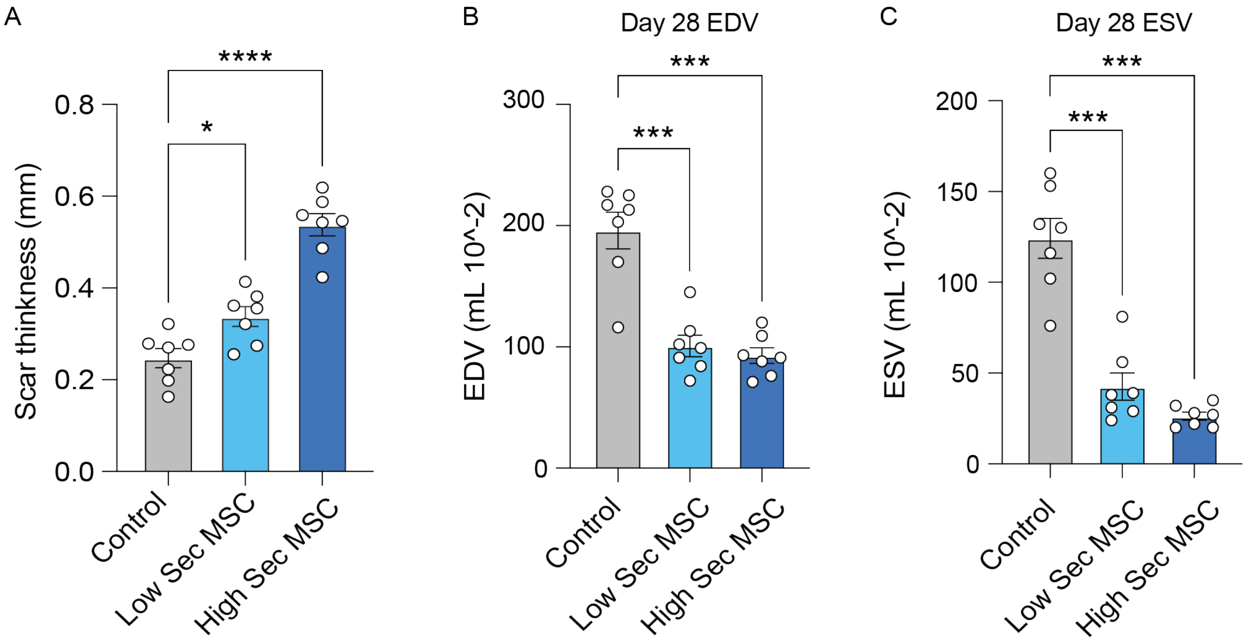

Supplementary Figure 9. High EV-secreting MSCs show augmented ability to mitigate cardiac remodeling after MI. A) Quantified left ventricle wall thickness of heart sections (n = 7, biological replicates). B) End-diastolic volume (EDV) of the control group, low or high EV secreting MSC treatment group at day 28 post MI. C) End-systolic volume (ESV) the control group, low or high EV secreting MSC treatment group at day 28 post MI. All data are means ± SEM. Comparisons between more than two groups were performed using the one-way analysis of variance (ANOVA), followed by Tukey's honestly significant difference (HSD) post hoc test. The comparisons between samples are indicated by lines, and the statistical significance is indicated by asterisks above the lines. *$p < 0.05$, ***$p < 0.001$, ****$p < 0.0001$.

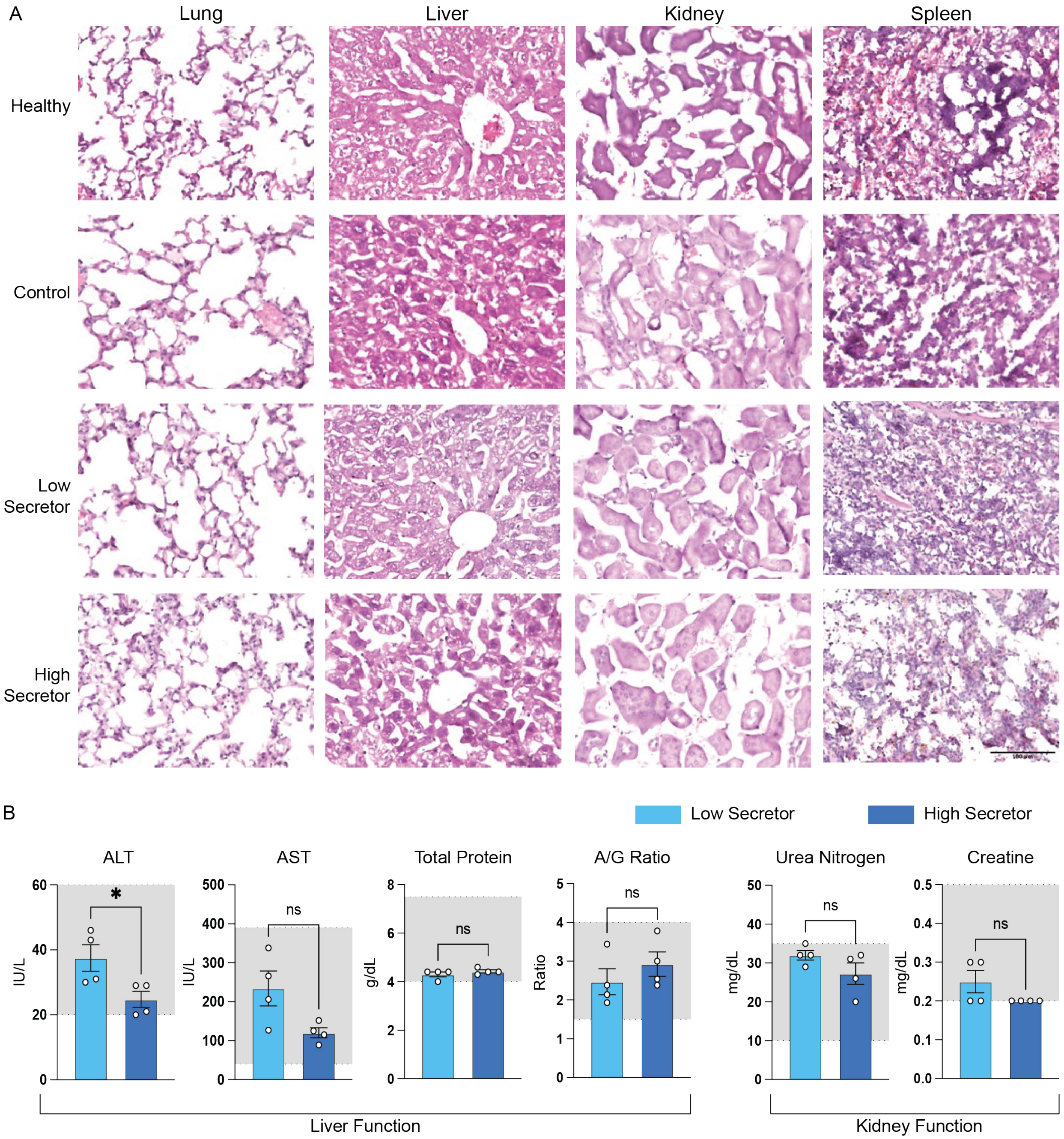

Supplementary Figure 10. Safety of high-sec and low-sec MSC treatment. A) H&E staining of major organs (heart, lung, liver, spleen, kidney) from mice of different treatment groups. Scale bar represents 100 μm. B) Serum chemistry from mice of different treatment groups. (Shaded region: range of normal values). Comparisons between two groups were performed with the unpaired, two-tailed Student's t-test, *$p < 0.05$, ns, not significant.

A

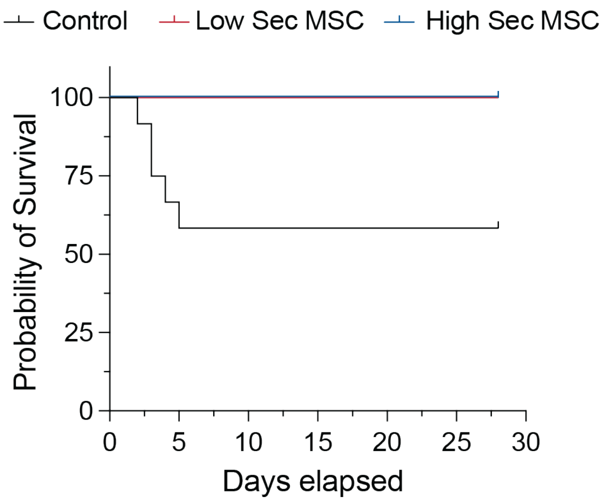

Supplementary Figure 11. Survival rate of mice treated with high and low EV secreting MSCs. A) Summary of survival rates of the control group (n=12), low or high EV secreting MSC treatment group (n=7). Low secreting MSC treatment group's survival curve (red) is overlapping with high secreting MSC treatment group curve (blue). The control group started with n=12 mice at day 0, with 7/12 surviving at 1 week post the induction of myocardial infarction. The dead animals were excluded from the following echocardiography study as they did not make it to the next time points for echocardiographic analysis (day 14 and day 28). The MSC treatment groups have n=7 from day 0 to day 28 as no death in these two groups were observed.

Supplementary Table 1. Individual electrocardiography data of the experimental mice.

		Baseline	D2	D14	D28
HighSec_1	EDV (ml 10^-2)	55	121	114	91
	ESV (ml 10^-2)	12	70	39	22
	LVEF (%)	77.8	42.5	66.1	76.4
	LVFS (%)	40.3	17.5	31.3	39.2
HighSec_2	EDV (ml 10^-2)	102	160	69	71
	ESV (ml 10^-2)	23	81	18	20
	LVEF (%)	77.6	49.4	74.3	70.7
	LVFS (%)	40.3	21.1	37.3	35.2
HighSec_3	EDV (ml 10^-2)	91	159	91	88
	ESV (ml 10^-2)	22	66	24	20
	LVEF (%)	76.4	59.1	73.8	77
	LVFS (%)	39.2	26.8	36.7	39.7
HighSec_4	EDV (ml 10^-2)	81	170	150	120
	ESV (ml 10^-2)	22	78	60	35
	LVEF (%)	73.4	54.4	60	70.6
	LVFS (%)	36.6	23.9	27.3	34.5
HighSec_5	EDV (ml 10^-2)	99	255	165	109
	ESV (ml 10^-2)	23	140	43	32
	LVEF (%)	76.7	44.9	74	71.1
	LVFS (%)	39.3	18.9	37.7	34.9
HighSec_6	EDV (ml 10^-2)	71	150	81	76
	ESV (ml 10^-2)	22	81	31	27
	LVEF (%)	69.1	45.8	61.8	64.8
	LVFS (%)	33.2	19.2	28.25	30.2
HighSec_7	EDV (ml 10^-2)	95	222	186	93
	ESV (ml 10^-2)	22	136	75	28
	LVEF (%)	76.2	38.8	59.9	63.4
	LVFS (%)	39	15.8	27.2	33.5
LowSec_1	EDV (ml 10^-2)	72	145	106	84
	ESV (ml 10^-2)	18	66	43	31
	LVEF (%)	75.4	54.8	64.6	63.2
	LVFS (%)	38.2	26.6	26.9	29.2
LowSec_2	EDV (ml 10^-2)	95	252	242	145
	ESV (ml 10^-2)	22	161	136	81
	LVEF (%)	77.3	35.7	43.7	44.2
	LVFS (%)	40	23.9	18.3	18.4
LowSec_3	EDV (ml 10^-2)	102	222	150	102
	ESV (ml 10^-2)	24	95	63	39
	LVEF (%)	76.1	57.3	58.2	62.2
	LVFS (%)	39	21.4	26.1	28.2
LowSec_4	EDV (ml 10^-2)	102	150	72	72

	ESV (ml 10^-2)	29	72	23	24
	LVEF (%)	71.4	52.3	67.9	65.8
	LVFS (%)	35.1	23.1	32.4	30.9
LowSec_5	EDV (ml 10^-2)	95	123	114	99
	ESV (ml 10^-2)	23	69	41	29
	LVEF (%)	75.8	44.1	64.3	70.3
	LVFS (%)	38.7	14.4	30	34.2
LowSec_6	EDV (ml 10^-2)	73	122	175	91
	ESV (ml 10^-2)	20	75	99	38
	LVEF (%)	72.1	38.7	43.7	57.9
	LVFS (%)	35.5	15.7	18.2	25.8
LowSec_7	EDV (ml 10^-2)	67	186	121	113
	ESV (ml 10^-2)	20	106	62	56
	LVEF (%)	69.5	42.9	48.7	50.7
	LVFS (%)	33.6	17.8	20.7	21.8
Control_1	EDV (ml 10^-2)	90	213	203	213
	ESV (ml 10^-2)	31	108	115	130
	LVEF (%)	65.3	49.4	43.1	38.9
	LVFS (%)	30.6	21.2	17.9	15.8
Control_2	EDV (ml 10^-2)	99	245	202	217
	ESV (ml 10^-2)	27	138	117	132
	LVEF (%)	72.7	43.6	42.1	39.1
	LVFS (%)	36.1	18.2	17.4	16
Control_3	EDV (ml 10^-2)	109	229	192	225
	ESV (ml 10^-2)	36	126	119	153
	LVEF (%)	67.2	45.2	37.8	32
	LVFS (%)	32	19	15.3	12.6
Control_4	EDV (ml 10^-2)	80	165	147	116
	ESV (ml 10^-2)	27	107	88	76
	LVEF (%)	66.7	35.3	40	35.1
	LVFS (%)	31.5	14.1	16.3	14
Control_5	EDV (ml 10^-2)	84	171	162	203
	ESV (ml 10^-2)	30	114	91	116
	LVEF (%)	64.1	33.1	43.8	42.7
	LVFS (%)	29.8	13.1	18.2	17.7
Control_6	EDV (ml 10^-2)	74	249	263	170
	ESV (ml 10^-2)	19	131	150	102
	LVEF (%)	74.2	47.3	42.9	40.1
	LVFS (%)	37.25	20.1	17.9	16.4
Control_7	EDV (ml 10^-2)	80	149	181	228
	ESV (ml 10^-2)	18	90	118	160
	LVEF (%)	77.5	39.7	34.8	29.8
	LVFS (%)	40.1	16.2	13.9	11.7
