# Supplementary material for: Optimizing cell therapy by sorting cells with high extracellular vesicle secretion
Source: Nat Commun. 2024 Jun 7;15:4870. doi: 10.1038/s41467-024-49123-1 (PMC11161503; doi:10.1038/s41467-024-49123-1)
Supplement: Supplementary file 2 — Reporting Summary [file 41467_2024_49123_MOESM2_ESM.pdf]

nature portfolio

Corresponding author(s): Dino Di Carlo
Ke Cheng

Last updated by author(s): 05/04/2024

Reporting Summary

Nature Portfolio wishes to improve the reproducibility of the work that we publish. This form provides structure for consistency and transparency in reporting. For further information on Nature Portfolio policies, see our Editorial Policies and the Editorial Policy Checklist.

Statistics

For all statistical analyses, confirm that the following items are present in the figure legend, table legend, main text, or Methods section.

n/a	Confirmed	
☐	☒	The exact sample size (*n*) for each experimental group/condition, given as a discrete number and unit of measurement
☐	☒	A statement on whether measurements were taken from distinct samples or whether the same sample was measured repeatedly
☐	☒	The statistical test(s) used AND whether they are one- or two-sided<br>*Only common tests should be described solely by name; describe more complex techniques in the Methods section.*
☒	☐	A description of all covariates tested
☐	☒	A description of any assumptions or corrections, such as tests of normality and adjustment for multiple comparisons
☐	☒	A full description of the statistical parameters including central tendency (e.g. means) or other basic estimates (e.g. regression coefficient) AND variation (e.g. standard deviation) or associated estimates of uncertainty (e.g. confidence intervals)
☐	☒	For null hypothesis testing, the test statistic (e.g. *F*, *t*, *r*) with confidence intervals, effect sizes, degrees of freedom and *P* value noted<br>*Give P values as exact values whenever suitable.*
☒	☐	For Bayesian analysis, information on the choice of priors and Markov chain Monte Carlo settings
☒	☐	For hierarchical and complex designs, identification of the appropriate level for tests and full reporting of outcomes
☒	☐	Estimates of effect sizes (e.g. Cohen's *d*, Pearson's *r*), indicating how they were calculated

Our web collection on statistics for biologists contains articles on many of the points above.

Software and code

Policy information about availability of computer code

Data collection	10X Genomics 3' Gene Expression
Data analysis	10X Genomics Cell Ranger Count v7.0.0 with reference (Human (GRCh38) 2020-A) from 10X cloud was used to analyze data. Software is available at https://www.10xgenomics.com/products/cloud-analysis. Further analysis was performed using 10X Genomics Loupe browser (cloupe, available at https://www.10xgenomics.com/products/chromium-analysis) for UMAP generation and differential gene expression.

For manuscripts utilizing custom algorithms or software that are central to the research but not yet described in published literature, software must be made available to editors and reviewers. We strongly encourage code deposition in a community repository (e.g. GitHub). See the Nature Portfolio guidelines for submitting code & software for further information.

Data

Policy information about availability of data

All manuscripts must include a data availability statement. This statement should provide the following information, where applicable:

- Accession codes, unique identifiers, or web links for publicly available datasets
- A description of any restrictions on data availability
- For clinical datasets or third party data, please ensure that the statement adheres to our policy

Data is available at GSE240981.

Research involving human participants, their data, or biological material

Policy information about studies with human participants or human data. See also policy information about sex, gender (identity/presentation), and sexual orientation and race, ethnicity and racism.

Reporting on sex and gender	This study did not involve human participants, their data or biological material.
Reporting on race, ethnicity, or other socially relevant groupings	This study did not involve human participants, their data or biological material.
Population characteristics	This study did not involve human participants, their data or biological material.
Recruitment	This study did not involve human participants, their data or biological material.
Ethics oversight	This study did not involve human participants, their data or biological material.

Note that full information on the approval of the study protocol must also be provided in the manuscript.

Field-specific reporting

Please select the one below that is the best fit for your research. If you are not sure, read the appropriate sections before making your selection.

[x] Life sciences [ ] Behavioural & social sciences [ ] Ecological, evolutionary & environmental sciences

For a reference copy of the document with all sections, see nature.com/documents/nr-reporting-summary-flat.pdf

Life sciences study design

All studies must disclose on these points even when the disclosure is negative.

Sample size	Sample size for in vivo study was based on our previously published study (Li, J. et al., Chemical Engineering Journal, 2022., Zhu, D. et al., Nature Communications, 2021., Zhu, D. et al., Eur Heart J, 2023.) and power calculation (power=0.9, effect size=0.9, significance level=0.05) which yielded that sample size of 7 is sufficient for analysis.
Data exclusions	No data was excluded in this study.
Replication	In the in vivo study, 7 biological replicates were conducted in each group and all attempts for these replicates were successful.
Randomization	In the in vivo study, groups were defined as mice (7 biological replicates) receiving high EV secreting MSC, low EV secreting MSC or no MSC embedded hydrogels. Mice were randomly allocated to each group.
Blinding	Blinding was not performed in our study to directly compare therapeutic efficacy between high or low EV secreting MSCs vs. no MSC treated groups. The individual preparing the cell populations also conducted the animal studies and so was not blinded.

Reporting for specific materials, systems and methods

We require information from authors about some types of materials, experimental systems and methods used in many studies. Here, indicate whether each material, system or method listed is relevant to your study. If you are not sure if a list item applies to your research, read the appropriate section before selecting a response.

Materials & experimental systems

n/a	Involved in the study	
[ ]	[x]	Antibodies
[ ]	[x]	Eukaryotic cell lines
[x]	[ ]	Palaeontology and archaeology
[ ]	[x]	Animals and other organisms
[x]	[ ]	Clinical data
[x]	[ ]	Dual use research of concern
[x]	[ ]	Plants

Methods

n/a	Involved in the study	
[x]	[ ]	ChIP-seq
[ ]	[x]	Flow cytometry
[x]	[ ]	MRI-based neuroimaging

Antibodies

Antibodies used	biotinylated anti-human CD63 (1:12.5, Biolegend, 353018), anti-human CD9 BV650 (1:20, BD, 743049), Rat anti-mouse CD9 PE (1:20, BD, 546234), rabbit anti-mouse Caspase 3 (1:100; ab184787, Abcam, Cambridge, UK), rabbit anti-mouse Ki67 (1:100; ab15580, Abcam), anti-mouse CD31 antibody (1:100; EPR17259, Abcam), mouse Anti-Cardiac Troponin T antibody (1:100; ab8295, Abcam)
Validation	All antibodies were purchased from commercial vendors and validated by the manufacturer. Antibody dilution used in nanovial assays were validated against purified EVs (anti-CD63, anti-CD9) to elicit staining in fixed percentages of nanovials with EV secretion signal and also based on previous studies (Udani, S. et al., Nature Nanotechnology 2023 1–10 (2023), Cheng, R.Y.H. et al., Nature Communications 2023 14:1 14, 1-15 (2023). The dilution of all other antibodies were followed based on the manufacturer's protocol. (https://www.abcam.com/products/primary-antibodies/caspase-3-antibody-epr18297-ab184787.html, https://www.abcam.com/products/primary-antibodies/ki67-antibody-ab15580.html, https://www.abcam.com/products/primary-antibodies/cd31-antibody-epr17259-ab182981.html, https://www.abcam.com/products/primary-antibodies/cardiac-troponin-t-antibody-1c11-ab8295.html)

Eukaryotic cell lines

Policy information about cell lines and Sex and Gender in Research

Cell line source(s)	ASC52telo, hTERT immortalized adipose derived mesenchymal stem cells SCRC-4000™ (ATCC), Mouse mesenchymal stem cells (Cell Biologics, C57-6043), HL-1 cells (Sigma, SCC065)
Authentication	Transcriptomic analysis authenticated the MSC lineage of the cell source;<br>Authenticated by ATCC: https://www.atcc.org/products/scrc-4000<br>Authenticated by Cell Biologics, Inc.: https://cellbiologics.com/index.php?route=product/product&path=2_47_101_394&product_id=22156<br>Authenticated by ATCC: https://www.atcc.org/products/crl-1446
Mycoplasma contamination	All cell lines tested negative for mycoplasma contamination, as indicated in the detailed product information provided by ATCC and Cell Biologics, Inc.
Commonly misidentified lines (See ICLAC register)	No misidentified lines were used.

Animals and other research organisms

Policy information about studies involving animals; ARRIVE guidelines recommended for reporting animal research, and Sex and Gender in Research

Laboratory animals	6-8-week old C57BL/6 mice (Charles River C57BL/6NCrl)
Wild animals	The study did not involve wild animals
Reporting on sex	Both female and male animals were included in this study. No sex- or gender-based analyses was performed in this study.
Field-collected samples	The study did not involve field-collected samples.
Ethics oversight	All studies and protocols were approved by the Institutional Animal Care and Use Committee (IACUC) of North Carolina State University (protocol: 19-811-B).

Note that full information on the approval of the study protocol must also be provided in the manuscript.

Plants

Seed stocks	*Report on the source of all seed stocks or other plant material used. If applicable, state the seed stock centre and catalogue number. If plant specimens were collected from the field, describe the collection location, date and sampling procedures.*
Novel plant genotypes	*Describe the methods by which all novel plant genotypes were produced. This includes those generated by transgenic approaches, gene editing, chemical/radiation-based mutagenesis and hybridization. For transgenic lines, describe the transformation method, the number of independent lines analyzed and the generation upon which experiments were performed. For gene-edited lines, describe the editor used, the endogenous sequence targeted for editing, the targeting guide RNA sequence (if applicable) and how the editor was applied.*
Authentication	*Describe any authentication procedures for each seed stock used or novel genotype generated. Describe any experiments used to assess the effect of a mutation and, where applicable, how potential secondary effects (e.g. second site T-DNA insertions, mosiacism, off-target gene editing) were examined.*

Flow Cytometry

Plots

Confirm that:

- [x] The axis labels state the marker and fluorochrome used (e.g. CD4-FITC).
- [x] The axis scales are clearly visible. Include numbers along axes only for bottom left plot of group (a 'group' is an analysis of identical markers).
- [x] All plots are contour plots with outliers or pseudocolor plots.
- [x] A numerical value for number of cells or percentage (with statistics) is provided.

Methodology

Sample preparation	Nanovials were resuspended in Dulbecco's Phosphate Buffered Saline (Thermo Fisher) with 0.05% Pluronic F-127 (Sigma), 1% 1X antibiotic-antimycotic (Thermo Fisher), and 0.5% bovine serum albumin (Sigma).
Instrument	SONY SH800S
Software	SONY Cell Sorter Software
Cell population abundance	In the post-sort sample, >95% of nanovials contained MSCs with secretion signal. The purity of sample was validated by taking images of samples on a fluorescence microscope.
Gating strategy	Nanovials were gated based on FSC-Height and SSC-Area first. Nanovials with live cells were gated based on Calcein AM (FITC) signal and ones with secretion signal were gated based on anti-CD9 BV650 Area vs. Height signal. All gates were created based on a negative control sample.

- [x] Tick this box to confirm that a figure exemplifying the gating strategy is provided in the Supplementary Information.
